# Practice of oxygen administration in patients hospitalized in internal medicine wards and intensive care units: A single-center prospective, observational study

**DOI:** 10.1590/1516-3180.2024.0323.29012025

**Published:** 2025-05-02

**Authors:** Saliha Bozkurt Esengul, Arzu Topeli, Burcin Halacli

**Affiliations:** I Department of Internal Medicine, Faculty of Medicine, Hacettepe University, Ankara, Türkiye.; II Division of Intensive Care Medicine, Department of Internal Medicine, Faculty of Medicine, Hacettepe University, Ankara, Türkiye.; III Division of Intensive Care Medicine, Department of Internal Medicine, Faculty of Medicine, Hacettepe University, Ankara, Türkiye.

**Keywords:** Inpatients, Inhalation, Disease progression, Oxygen saturation, Respiratory failure, Oxygen therapy, COPD

## Abstract

**BACKGROUND:**

Oxygen is widely used to treat hypoxemia.

**OBJECTIVE:**

To determine the frequency of inappropriate oxygen administration in patients admitted to Internal Medicine (IM) wards and intensive care units (ICU).

**DESIGN AND SETTING:**

Single-center prospective, observational study in a tertiary university hospital in Ankara, Türkiye.

**METHODS:**

Patients who were hospitalized in the IM wards and ICU and were receiving oxygen were recruited. Every 6 hours, the oxygenation parameters were noted, and the averages over the first 24 hours of oxygen usage were recorded. Inappropriate usage was defined as oxygen flow rates > 6 L/min in the nasal cannula and < 5 L/min and > 10 L/min in the simple face mask, application of the simple face mask in chronic obstructive lung disease (COPD) exacerbation, SpO_2_ > 98% in general, or SpO_2_ > 92% in COPD exacerbation.

**RESULTS:**

Of the 397 patients, 20% in the IM wards and 50% of 124 in the ICU received oxygen. The oxygen method used was nasal cannula in 51%, simple face mask in 21%, and high-flow nasal cannula in 4% of the patients. Among the simple face mask applications, 46% were < 5 L/min and 5% were > 10 L/min. Among the 62% of patients with COPD exacerbations, the SpO_2_ was > 92%.

**CONCLUSION:**

The frequency of oxygen use was 20% among patients hospitalized in IM wards and 50% among patients in the ICU. Almost half of the simple face mask applications were inappropriate.

## INTRODUCTION

Oxygen is frequently used to treat hypoxemia and prevent and treat hypoxia.^1^ Oxygen support is provided by low- or high-flow systems according to the underlying medical condition and tolerance of the patient. Low-flow systems have an oxygen flow rate that is less than the inspiratory flow rate (15–20 L/min), which causes room air to be mixed with supplemental oxygen. Because the ratio in this mixture is affected by the patient’s minute ventilation, the fraction of inspired oxygen (FiO_2_) varies. Nasal cannulas, simple face masks, non-rebreather and partial rebreather masks with reservoirs, diffuser masks, and transtracheal catheters are among the systems that deliver oxygen at a low flow rate. The nasal cannula is used in mild hypoxemia at flow rates of 1–6 L/min, and supplementation of 1 L/min oxygen increases the oxygen fraction (FiO_2_) by 4%.^2^ A flow rate > 6 L/min does not increase FiO_2_, whereas it can cause negative effects such as mucosal drying and disruption of ciliary activity. A simple face mask creates 40–60% FiO_2_.^1^ These masks should not be used with flow rates < 5 L/min due to the risk of carbon dioxide (CO_2_) rebreathing.^2,3^ Thus, due to the risk of hypercapnia, it should not be preferred in patients with hypercapnia or hypercapnia risk such as chronic obstructive lung disease (COPD).^1^

Similar to any medical intervention, it is important to establish a specific oxygen support range that prevents hypoxia while avoiding the risk of hyperoxia, which has drawbacks. It has been shown that hyperoxia causes increased systemic vascular resistance,^4^ oxygen-induced hypercapnia,^5^ resorption atelectasis,^6^ oxidative stress,^7,8^ pulmonary toxicity,^9^ neurotoxicity and ischemia reperfusion injury.^10,11^ In addition, there are studies showing that in-vitro exposure of human leukocytes to more than 80% FiO_2_ increases cytokine production,^12^ as well as decreases the antimicrobial activity of alveolar macrophages.^13^

The British Thoracic Society (BTS) recommends maintaining a peripheral oxygen saturation (SpO_2_) level of 94–98% in adult patients. However, if there is concern about hypercapnia, a target range of 88–92% is recommended. Alongside oxygen saturation, another parameter for oxygenation is the ratio of arterial oxygen partial pressure and the fraction of inspired oxygen (PaO_2_/FiO_2_).^14^ Clinical studies have identified a positive correlation between the ratios of PaO_2_/FiO_2_ and SpO_2_/FiO_2_, prompting the consideration of using the latter measurement as an alternative due to its non-invasive nature.^15,16^

## OBJECTIVE

This study primarily aimed to determine the frequency of oxygen use among patients in Internal Medicine (IM) wards and intensive care units (ICU) and examine instances of improper oxygen utilization. The secondary objectives of this study were to delineate methods of oxygen support, categorize patients into groups according to their SpO_2_ levels (< 94%, 94%–98%, and > 98%), and to explore the relationship between the ratios of SpO_2_/FiO_2_ and PaO_2_/FiO_2_.

## METHODS

This was a prospective observational study carried out between 01-31 March 2022 among patients hospitalized in the Department of Internal Medicine wards (220 beds) and the ICU (20 beds) of a University Hospital. Patients aged ≥18 years who received oxygen support were included in this study. Pregnant patients and those with acute myocardial infarction, stroke, or carbon monoxide intoxication were excluded.

Patient demographics, comorbidities, reasons for hospitalization, and need for oxygen were recorded. Oxygen support data were obtained from patient charts at 6-hour intervals (four quartiles) within the first 24 hours of enrollment. To avoid surveillance bias, the average of the 6-hour oxygenation data was calculated and recorded, and the 24-hour average was then calculated. We recorded oxygen support methods such as nasal cannula, simple face mask, high-flow nasal oxygen (HFNO), and mechanical ventilation (MV), as other methods were not used during the study period. We recorded the oxygen flow rate using a nasal cannula and a simple face mask. Using HFNO and MV, we recorded FiO_2_ values. The SpO_2_ values of all the patients were obtained. If available, we also obtained the values of PaO_2_ and SaO_2_ from the hospital’s electronic medical record system. Using these data, we calculated PaO_2_/FiO_2_ and SpO_2_/FiO_2_ ratios. The ROX index, defined as the ratio of PpO_2_/FIO_2_ to the respiratory rate, was calculated for all patients. The modified early warning score (MEWS) was evaluated to provide information on the physiological state of ward patients. In ICU patients, the Acute Physiology and Chronic Health Evaluation (APACHE) II score was calculated using the worst data from the first 24 hours of ICU admission. All patients were followed up for 3 months for the development of infection.

In patients receiving nasal oxygen, FiO_2_ was calculated using the following formula: FiO_2_ = 21% + (4xO_2_ flow L/min). We used an approximate FiO_2_ value based on the oxygen flow rate in patients using a simple face mask.^2^ In the case of HFNO and MV use, we obtained the delivered FiO_2_ value from the patient chart.

Among the oxygen support methods, the use of nasal oxygen at a rate > 6 L/min and the use of a simple face mask with a flow rate < 5 L/min and > 10 L/min were labeled inappropriate. We also designated simple face mask use as inappropriate for patients with COPD exacerbation because of the risk of hypercapnia. Additionally, oxygen use that exceeded the target SpO_2_ levels of 88–92% and > 98% in patients with and without COPD exacerbation, respectively, as per the BTS guideline, was labeled as inappropriate usage. All ‘inappropriate usage’ data are expressed according to 6-hour interval records (i.e., four records per patient) rather than patient numbers to prevent surveillance bias.

Based on the BTS guideline recommendation of maintaining SpO_2_ between 94% and 98% in adult patients, we divided our patients into three groups according to their SpO_2_ values: < 94%, 94–98%, and > 98%.

### Statistical analysis

Normally distributed numerical variables are presented as mean ± standard deviation (SD) and others as median (interquartile range-IQR). Normally distributed variables were compared using Student’s *t*-test, and others were compared using the Mann-Whitney U test. The chi-square and Fisher’s exact tests were used to compare categorical variables. Variance analysis of parametric variables was performed using one-way analysis of variance (ANOVA) test. The homogeneity of variances was evaluated using Levene’s test. The Kruskal-Wallis test was performed for the variance analysis of nonparametric variables. Post-hoc pairwise comparisons were performed using the Mann-Whitney U test and evaluated using Bonferroni correction. Correlation coefficients and statistical significance of the relationships between variables were determined using the Spearman test for cases in which at least one of the variables was not normally distributed. Statistical significance was defined as a two-tailed P-value < 0.05. SPSS software (IBM, Armonk, New York, United States) version 25 was used for the statistical analysis.

### Ethical considerations

This study was approved by the Ethics Board of Hacettepe University Non-Interventional Clinical Researchers (date: 12/07/2021, No: GO 21/1094). The procedures followed in this study were performed in accordance with the principles of the Declaration of Helsinki. Informed consent was obtained from patients or legal surrogate decision-makers among those who met the study criteria.

## RESULTS

**Figure 1:**
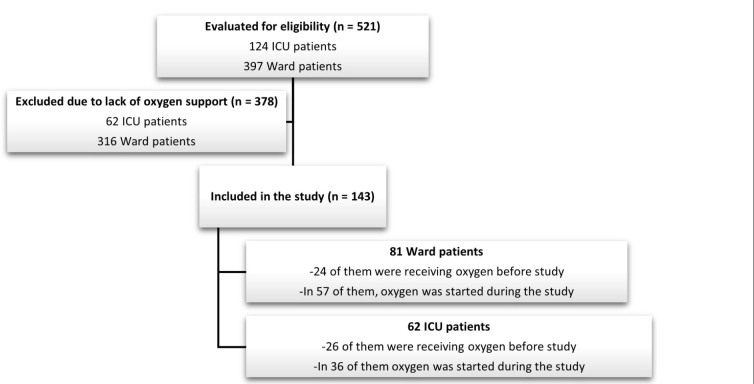
Flowchart of the study.

Among 397 ward and 124 ICU patients, 143 (27%) patients who were receiving oxygen were included in the study. Eighty-one (57%) patients were from the ward and 62 (43%) were from the ICU. During the one-month study period, 50 (35%) patients were on oxygen before the beginning of the study. The remaining patients were recruited on the first day of oxygen support (Figure 1).

ICU = intensive care unit.

The three most common comorbidities were hypertension, solid malignancy, and chronic pulmonary disease (Table 1). The most common reason for hospitalization was infection (31%). Oxygen demand developed in 98 (69%) patients because of respiratory failure. Thirteen (9%) patients received oxygen because of hypercapnic respiratory failure. The median duration of oxygen therapy received by the patients was 15 (6–33) days. The overall median length of hospitalization for all patients was 28 days (16–55), and the median duration of stay in the ICU was 13 (7–29) days. Patients in the wards had a median hospital stay of 26 days (14–49). Notably, 20% of the ward patients who received oxygen required ICU admission during follow-up.

**Table 1:** Baseline characteristics of the patients

Characteristics	All subjects (n = 143)
**Age, mean ± SD, years**	65 ± 17
**Sex, n (%)**	
Male	79 (55.0)
Female	64 (45.0)
**Comorbidities, n (%)**	
Hypertension	63 (44.1)
Solid malignancy	62 (43.4)
Chronic pulmonary disease	50 (35.0)
Diabetes mellitus	43 (30.1)
Coronary artery disease	36 (25.2)
Hematological malignancy	28 (19.6)
Chronic kidney disease	25 (17.5)
Thyroid diseases	21 (14.7)
Heart failure	15 (10.5)
Cerebrovascular events	10 (7.0)
Connective tissue diseases	10 (7.0)
Dementia	7 (4.9)
GI diseases	3 (2.1)
Immunodeficiency	2 (1.4)
**Initial reason for hospitalization, n (%)**	
Infection	45 (31.0)
Chemotherapy administration	27 (19.0)
Hypervolemia	10 (7.0)
Hypercapnic respiratory failure	11 (8.0)
Hypoxemic respiratory failure	10 (7.0)
Sepsis	7 (5.0)
GI bleeding	5 (3.5)
Others	28 (19.5)
**Reason for oxygen support, n (%)**	
Respiratory failure	98 (69.0)
Hypoxemic respiratory failure	85 (59.0)
Hypercapnic respiratory failure	13 (9.0)
Hypervolemia	22 (15.0)
Sepsis/Septic shock	18 (13.0)
Others	5 (4.0)
Duration of oxygen support, *days*	15 (6–33)
Length of hospital stay, *days*	28 (16–55)
Length of ICU stay, *days*	13 (7–29)
Length of ward stay, *days*	26 (14–49)

SD = standard deviation; GI = Gastrointestinal; ICU = intensive care unit.

We obtained 572 oxygen data points, including the support method from the 6-hour intervals of each of the 143 patients. Accordingly, 51% of the patients used nasal oxygen, 21% used a simple face mask, 18% used invasive MV (IMV), 6% used non-invasive MV (NIMV) and 4% used HFNO. Nasal oxygen was predominantly administered outside the ICU in the IM wards, whereas HFNO, NIMV, and IMV were predominantly administered in the ICU, as seen in Table 2.

**Table 2:** Oxygen support methods according to 6-hour interval records

Oxygen and respiratory support methods n (%)				
	All administrations (n = 572)	Ward (n = 324)	ICU (n = 248)	P value
Nasal cannula	291 (51)	239 (74)	52 (21)	**< 0.001**
Simple face mask	117 (21)	63 (19)	54 (22)	0.49
IMV	102 (18)	8 (2)	94 (38)	**< 0.001**
NIMV	37 (6)	13 (4)	24 (10)	**0.006**
HFNO	25 (4)	1 (0.3)	24 (10)	**< 0.001**

ICU = intensive care unit; IMV = invasive mechanical ventilation; NIMV = non-invasive mechanical ventilation; HFNO = high-flow nasal oxygen.

All 291 nasal oxygen administrations were performed at ≤ 6 L/min. However, among the 117 simple facemask administrations, 54 (46%) had flow rates of < 5 L/min and 6 (5%) had flow rates of > 10 L/min. We labeled simple face mask usage as inappropriate for COPD exacerbation due to the risk of hypercapnia. Thirteen patients received oxygen therapy for COPD exacerbation. Among the 52 recordings in the 6-hour interval oxygen delivery method in 13 patients, eight (15%) had simple face masks (Table 3). Among patients with and without COPD exacerbation, eight (62%) and seven (5%) patients had saturations above the target range (Table 3).

**Table 3:** Inappropriate usage of oxygen according to 6-hour interval records

Inappropriate usage of simple face mask (According to flow rate) *n (%)*			
All administrations (n = 117)	Patients in the ward (n = 63)	Patients in ICU (n = 54)	P value
60 (51)	33 (52)	27 (50)	.79
Inappropriate usage of simple face mask in patients with COPD exacerbation (*n = 52) n (%)			
8 (15)			
**Inappropriate usage of oxygen support (According to SpO**_2_ **upper target limit)** *n (%)*			
**Patients with COPD exacerbation (n = 13)**		**Patients without COPD exacerbation (n = 130)**	
8 (62)		7 (5)	

COPD = chronic obstructive pulmonary disease; *All administrations in 6-hour intervals

According to the BTS guidelines, eight (62%) of 13 COPD exacerbation patients had saturation > 92% (target range of 88–92%), whereas seven patients (5%) without COPD exacerbation had saturation > 98% (target range of 94–98%) (Table 3).

**Figure 2:**
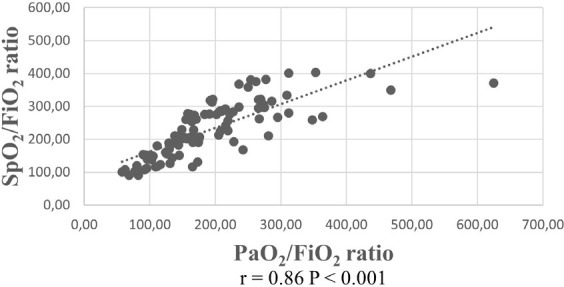
SpO_2_/FiO_2_-PaO_2_/FiO_2_ correlation.

A total of 117 arterial blood gas (ABG) analyses were performed on the patients at different 6-hour intervals. All SaO_2_ values of these 117 ABGs were ≤ 97%. As only 17% of patients underwent ABG testing within the ward setting, we performed a correlation analysis of SpO_2_/FiO_2_ and PaO_2_/FiO_2_ with 97 ABG analyses of ICU patients. We found a strong positive correlation between PaO_2_/FiO_2_ and SpO_2_/FiO_2_ (r = 0.86, P < 0.001; Figure 2).

The correlation was analyzed using 97 arterial blood gas analysis results obtained from patients in the intensive care unit.

All patients were grouped according to SpO_2_ values as per the BTS guideline cut-off values, and the descriptive statistics are listed in Table 4.

**Table 4:** Baseline characteristics of the patients grouped according to oxygen saturation

Parameters	SpO_2_<94% (n = 66)	SpO_2_94–98% (n = 70)	SpO_2_>98% (n = 7)	P value
**Age, years**	66 ± 16	64 ± 17	67 ± 16	0.55
**Sex (M/F)**	59/41	53/47	55/45	0.99
**Comorbidities, n (%)**				
COPD	15 (23)	14 (20)	2 (29)	0.83
Solid malignancy	24 (36)	35 (50)	3 (43)	0.27
Hematologic malignancy	10 (15)	15 (21)	3 (43)	0.18
**Reason for oxygen support, n (%)**				
Respiratory failure	48 (73)	46 (66)	4 (57)	0.12
Hypervolemia	12 (18)	10 (14)	0 (0)	0.42
Sepsis/Septic shock	4 (6)	11 (16)	3 (43)	0.12
Others	2 (3)	3 (4)	0 (0)	0.12
**Oxygen and respiratory support methods, n (%)**				
Nasal cannula	33 (50)	31 (44)	3 (43)	0.78
Simple face mask	16 (24)	21 (30)	0 (0)	0.21
IMV	9 (14)	13 (19)	3 (43)	0.14
NIMV	2 (3)	2 (3)	1 (14)	0.28
HFNO	6 (9)	3 (4)	0 (0)	0.40
**Cases of inappropriate usage of simple face mask/All simple face mask administrations, n (%)**	10/16 (62)	10/21 (48)	0 (0)	0.36
**ROX index**	11 ± 4	13 ± 4	15 ± 4	0.29
-Respiratory rate/min	23 ± 3	22 ± 3	22 ± 4	0.44
-SpO_2_ mean values	92.3 ± 1.3	95.4 ± 1	98.8 ± 0.6	**< 0.001**
**MEWS**	6 (5–8)	4 (4–6)	5 (4–6)	**< 0.001**
**APACHE II**	18 (15–23)	18 (13–22)	21 (19–25)	0.27
**Presence of infection at 3-month follow-up, n (%)**	37 (56)	40 (57)	5 (71)	0.73

COPD = chronic obstructive pulmonary disease; IMV = invasive mechanical ventilation; NIMV = non-invasive mechanical ventilation; HFNO = high-flow nasal oxygen; ROX = respiratory rate-oxygenation index; MEWS = modified early warning score; APACHE II = acute physiology and chronic health evaluation II.

## DISCUSSION

This study revealed that oxygen was administered to 20% of the patients in the IM wards and 50% in the ICU at a tertiary care university hospital. We observed that simple face masks were inappropriately used in approximately half of the instances. Furthermore, more than 50% of patients maintained SpO_2_ values > 94%. Additionally, more than half of the patients with COPD exacerbation had SpO_2_ levels > 92%. Importantly, there was a strong positive correlation between the SpO_2_/FiO_2_ and PaO_2_/FiO_2_ ratios.

Observational studies carried out during patients’ hospital admission or over a two-week period in the wards revealed that oxygen was administered to 10–31% of patients.^17,18^ It is important to note that these studies did not include patients who underwent NIMV and IMV. In a separate observational study conducted at a single center, Devoe et al. investigated patients receiving oxygen in both general wards and ICUs on two different non-consecutive days in 2019.^19^ Among the 745 patients admitted to surgical and medical wards, 116 (16%) received oxygen support, and among the 66 patients in the ICU, 37 (56%) required oxygen. Notably, this study also included patients who received respiratory assistance through NIMV and IMV. In our study, we prospectively recruited patients who required any type of oxygen support, including nasal cannula, simple face mask, HFNO, NIMV, and IMV, during the one-month period. We observed that 27% of patients used oxygen. When analyzed separately, we observed that 20% of patients in the IM wards and 50% of patients in the ICUs required oxygen. In Devoe’s study, among 153 patients who required oxygen support, 128 (83%) received a nasal cannula, 21 (14%) received oxygen via IMV, 3 (2%) received HFNO, and 1 (1%) received NIMV.^19^ In our study, a nasal cannula was applied in 51% of patients, simple face mask in 21%, IMV in 18%, NIMV in 6%, and HFNO in 4%. As expected, nasal cannulas were applied more frequently in the wards, whereas HFNO and IMV were applied more frequently in the ICUs.

However, few studies have investigated the appropriateness of oxygen therapy. In a study conducted by Ballance et al.^20^ investigating the level of knowledge about the basic principles of oxygen support systems using a questionnaire administered to 46 physicians, answers to questions regarding the nasal cannula, simple face mask, Venturi mask, and non-rebreather mask with reservoir were correct at 100%, 93%, 96%, and 91%, respectively. In a study investigating the oxygen management of postoperative patients, 46 patients were administered oxygen with a simple face mask, and six (37%) of them received a flow rate of < 5 L/min.^21^ In our study, nasal oxygen was not applied at flow rates > 6 L/min. However, simple face masks were used inappropriately, with flow rates of < 5 L/min in 46% and > 10 L/min in 5% of administrations.

In current guidelines, there is no consensus on the lower and upper limits of oxygen support. The BTS recommends maintaining oxygen saturation of 94–98% in adult critically ill patients if there is no risk of hypercapnia but states that further studies should be performed for a more ideal target range, for example, 92–96%. In other conditions with the risk of COPD and hypercapnic respiratory failure, the target saturation is accepted as 88–92%.^1^ The British Medical Journal (BMJ) recommends that oxygen support should be given to adult critically ill patients with a saturation target of < 96%.^22^ The Australian and New Zealand Thoracic Society has determined the target saturation as 92–96% in acute oxygen use in adult patients.^23^ In the Global Initiative for Chronic Obstructive Lung Disease (GOLD) guideline, the saturation target in COPD exacerbation is 88–92%.^24^

Suzuki et al.^25^ investigated oxygen administration in patients undergoing MV and recorded the oxygenation data of 51 patients with a total of 358 MV days at 6-hour intervals in order to prevent surveillance bias, similar to our study. SpO_2_ was > 98% in 59% of patients. In the study by Devoe et al.,^19^ SpO_2_ was > 96% in 84 (55%) patients and < 90% in only 2 (1%) patients. In addition, it was > 96% in all patients with COPD exacerbation. Owing to the absence of standardized guidelines, various clinical trials have adopted varying low and high oxygenation targets. In a randomized clinical trial conducted by van der Wal and colleagues,^26^ they examined mortality rates in mechanically ventilated ICU patients with different oxygenation targets were examined. They considered SpO_2_ levels of 91–94% as low oxygenation and SpO_2_ levels of 96–100% as high oxygenation. Their findings revealed that the median SpO_2_ values were 95% (IQR 94–97) for the low-oxygenation group, which was higher than the initial target, and 99% (IQR 98–100) for the high-oxygenation group. Our study revealed that SpO_2_ levels exceeded 94% for more than half of the patients. In line with the previously mentioned research, only 3% of the patients had saturation levels < 90%, and 13% had saturation levels < 92%. In contrast to the BTS guidelines, more than half of the patients with COPD exacerbation exhibited saturation levels > 92%. The most recent revision of the BTS guidelines was in 2017. Based on the premise of our study, we posit that the target range of 94–98% saturation might be on the higher side.

The SpO_2_/FiO_2_ ratio, which can be measured noninvasively, has been explored as an alternative to the PaO_2_/FiO_2_ ratio for monitoring oxygen support. For instance, Rice et al.^15^ investigated the SpO_2_/FiO_2_ ratio as a potential surrogate for the PaO_2_/FiO_2_ ratio in defining ARDS and discovered a positive correlation between the two (P < 0.001; r = 0.89). Pandharipande et al.^16^ also delved into the use of SpO_2_/FiO_2_ instead of the PaO_2_/FiO_2_ ratio in the SOFA score and observed a positive correlation between these ratios (r = 0.85, P < 0.001). In our study, a robust correlation between the SpO_2_/FiO_2_ and PaO_2_/FiO_2_ ratios was evident in patients admitted to the ICU.

The current study has certain limitations. Its single-center nature and relatively small sample size hinder the broad applicability of the findings. This was a cross-sectional study, with monitoring of oxygenation parameters restricted to the initial 24-hour period. This limitation affects the ability to identify inappropriate usage and long-term consequences of oxygen administration in patients with both low and high oxygen saturation levels. The sample size did not permit in-depth analysis within specific SpO_2_ subgroups or distinct disease categories (e.g., COPD). Unfortunately, owing to the limited number of patients (only 17%) undergoing ABG testing within the ward setting, we performed a correlation analysis of SpO_2_/FiO_2_ and PaO_2_/FiO_2_ on a smaller dataset involving ABG analyses exclusively from ICU patients. The assessment of oxygen support primarily relies on SpO_2_ values because it is non-invasive. While most hyperoxemia studies in the existing literature are based on PaO_2_ values, there is a requirement for investigations centered on saturation measurements to swiftly and noninvasively identify hyperoxemia.

## CONCLUSION

This study demonstrated that the frequency of oxygen use was 27% among all patients admitted to the Internal Medicine Department, with a 20% rate in the wards and 50% in ICUs at a tertiary care university hospital. Almost 50% of instances of simple face mask use constituted improper utilization. The SpO_2_ values were > 94% in more than half of the patients using oxygen and > 92% in more than half of the patients with COPD exacerbation. The frequent utilization of oxygen has underscored the necessity of examining the improper use of this readily available therapeutic option in a large dataset. There is also a requirement for research aimed at examining lower or potentially ideal cut-off values. Furthermore, the robust positive correlation observed between the SpO_2_/FiO_2_ and PaO_2_/FiO_2_ ratios in ICU patients suggests that SpO_2_/FiO_2_ could be a promising alternative ratio for facilities lacking arterial blood gas equipment or when invasive measurements are not preferred.
